# Genetic and functional characterization of the NanA sialidase from *Clostridium chauvoei*

**DOI:** 10.1186/1297-9716-42-2

**Published:** 2011-01-11

**Authors:** Edy M Vilei, Anders Johansson, Yvonne Schlatter, Keith Redhead, Joachim Frey

**Affiliations:** 1 Institute of Veterinary Bacteriology, Vetsuisse Faculty, University of Bern, Bern, Switzerland; 2 National Food Administration, Uppsala, Sweden; 3 Intervet/Schering-Plough Animal Health, Milton Keynes, UK

## Abstract

*Clostridium chauvoei *is the causative agent of blackleg, a wide spread serious infection of cattle and sheep with high mortality. In this study we have analyzed the sialidase activity of the NanA protein of *C. chauvoei *and cloned the sialidase gene *nanA*. Sialidase is encoded as a precursor protein of 722 amino acids with a 26 amino acid signal peptide. The mature sialidase has a calculated molecular mass of 81 kDa and contains the carbohydrate binding module 32 (CBM32, or F5/8 type C domain), the sialic acid binding module CBM40 and the enzymatically active sialidase domain found in all pro- and eukaryotic sialidases. Sialidase activity does not require the CBM32 domain. The NanA protein is secreted by *C. chauvoei *as a dimer. The *nanA *gene was found to be conserved and sialidase activity was found in *C. chauvoei *strains isolated over a period of 50 years from various geographical locations. Antiserum directed against a recombinant 40 kDa peptide containing CBM40 and part of the enzymatically active domain of NanA neutralized the secreted sialidase activity of all *C. chauvoei *strains tested.

## Introduction

*Clostridium chauvoei *is an anaerobic, endospore-forming Gram-positive bacterium known to be the causative agent of blackleg, a severe disease with high mortality affecting young cattle and sheep of any age. While *C. chauvoei *infections are specifically found in ruminants, a case of human fulminant gas gangrene caused by *C. chauvoei *was recently described [1]. Ingestion of *C. chauvoei *spores is probably the most common form of exposure and infected ruminants do not directly transmit the disease to other animals. The endospores of *C. chauvoei *can lie dormant in the soil for years and, after ingestion by the animal, they are assumed to cross over the gastro-intestinal tract, enter the bloodstream and finally migrate in various organs and muscles, where they remain dormant until stimulated to cause disease [2]. These spores are only activated in a low oxygen environment, such as that of damaged or bruised tissue in cattle. In sheep, blackleg is frequently associated with wounding, such as at shearing cuts, tail docking and castration site [3]. Once these stimulating events occur, the spores in the infected tissue germinate and multiply into the disease-causing *C. chauvoei *bacteria. Then, the disease progresses rapidly, with the infected animal dying in 12 to 36 h after the appearance of the first symptoms [4]. The molecular mechanisms of blackleg are not clearly understood. Due to the peracute evolution with severe oedematous lesions restricted to the local area, fever, loss of appetite, lameness and depression observed in blackleg outbreaks, it is speculated that virulence of *C. chauvoei *is caused by rapid spread of the activated, vegetative form of the bacterium in the infected tissue, followed by the production of potent toxins [4,5]. Although sialidases and toxins produced by *C. chauvoei *are believed to play a significant role in the spread of the pathogen and in the induction of lesions in affected tissues [5], they have not yet been characterized at the molecular and genetic level. It has to be mentioned here that, to date, only a few DNA sequences of *C. chauvoei *have been deposited in DDBJ/EMBL/GenBank databases including the 16S and the 23S rRNA genes, the 16S-23S rRNA spacer region, and the flagellin gene [6,7].

**Figure 1:**
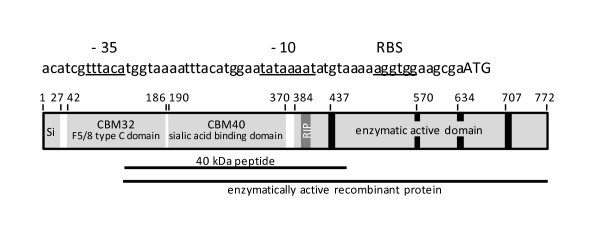
**Schematic representation of the NanA sialidase of *C. chauvoei***. Upper part: DNA sequence of the promoter region of *nanA *of strain JF4135 with -35 and -10 boxes and the ribosome binding site (RBS) underlined. The translational start codon (ATG) is indicated by upper case letters. Lower part: main domains of the NanA polypeptide. Numbers refer to amino acid positions and black bars represent the characteristic "Asp-boxes". Si is the signal sequence and RIP indicates the position of the first Arg residue of the catalytic triad with the characteristic Arg-Ile-Pro sequence. The horizontal lines indicate the part of NanA that was expressed as a recombinant 40 kDa polyhistidine-tailed peptide to produce monospecific polyclonal antibodies in rabbits (upper) and the part of NanA expressed as enzymatically active recombinant protein (lower).

Sialidases, or neuraminidases (EC 3.2.1.18), are enzymes that cleave N-acetylneuraminic acid from carbohydrate polymers, such as mucin, glycoproteins, gangliosides and other sialoglycoconjugates, located on many mammalian cell membranes. The mature sialidase is generally composed of three main domains (Figure 1). At its N-terminal end, it contains the F5/8 type C domain, alternatively designated CBM32 (carbohydrate binding module 32) or discoidin domain, that appears to be primarily involved in binding of terminal galacto-configured sugar residues and is also found in the *C. perfringens *sialidase NanJ [8]. The central domain contains a CBM40 module (or lectin-like sialic acid binding domain) that was shown to be specific for binding sialic acid residues in NanJ of *C. perfringens *[8]. The enzymatically active site of sialidases is in the C-terminal end that contains the usual catalytic residues that are common to all sialidases [9-11], including a catalytic nucleophile tyrosine, an arginine triad that interacts with the carboxylate group of sialic acid, a tryptophan residue that forms a weak H-bond with the glycerol side chain of sialic acid, and an aspartic acid that forms H-bonds with the four OH-groups of sialic acid. Furthermore, the enzymatically active domain contains four "Asp-boxes" [10] characterized by the motif Ser-X-Asp-X-Gly-X-Thr-Trp, and the first arginine of the catalytic triad of sialidases generally belongs to a RIP (Arg-Ile-Pro) motif [12].

Sialidases have been found in viruses, bacteria, protozoa, fungi and metazoans [13]. In viruses, parasites and bacteria, sialidases are thought to be important for survival and pathogenicity of the organisms [14]. It has been established that viral sialidases have a central role in the pathogenesis of influenza [14,15]. The importance of sialidase in respiratory tract infections has been demonstrated for two bacterial species: *Streptococcus pneumoniae *and *Pseudomonas aeruginosa*. The neuraminidases (sialidases) NanA and NanB of *S. pneumoniae *have been shown to be essential for successful colonization of both upper and lower respiratory tract and for pneumococcal survival in the blood using an outbred MF1 mouse or chinchilla model [16,17]. The sialidase produced by *P. aeruginosa *has been shown to be a critical factor for colonization of the upper respiratory tract. It has also been shown that viral neuraminidase inhibitors blocked biofilm production and the bacterial colonization by *P. aeruginosa *[18]. Recently, Mally and colleagues [19] demonstrated the impact of sialidase in *Capnocytophaga canimorsus*, a commensal canine bacterium of the oral flora, that can cause severe septicaemia and meningitis in humans [20]. Wild type *C. canimorsus *is dependent on a surface exposed sialidase for growth and persistence in a murine infection model, whereas a sialidase deficient *C. canimorsus *mutant was unable to grow and persist in mice [19]. Sialidase may have an important role in blackleg by degrading tight junctions upon cleavage of sialic acids at a high rate, thus allowing the bacterium to spread through host tissue [5]. A sialidase activity has been reported in *C. chauvoei *[21], but the sialidase enzyme has not yet been characterized in detail [22,23]. Furthermore, the molecular and genetic basis of sialidase in *C. chauvoei *is still unclear.

This study reports the cloning of the *nanA *sialidase gene from *C. chauvoei*, its expression and the characterization of the sialidase NanA protein. Under native conditions in bacterial culture medium, *C. chauvoei *sialidase is found as a 150 kDa protein, in the form of a dimer of two NanA peptides with an apparent molecular mass of 72 kDa.

## Materials and methods

### Strains and culture conditions, DNA extraction and sequence analysis

The seven *C. chauvoei *strains used in this study are listed in Table 1. The *C. chauvoei *strains were grown anaerobically on Tryptic Soy Agar (TSA) medium containing 5% sheep erythrocytes (bioMérieux, Geneva, Switzerland) or in TGY broth (3% tryptic soy broth (Becton Dickinson AG, Allschwil, Switzerland), 2% glucose (Sigma-Aldrich chemical, St. Louis, MO, USA), 1% yeast extract (Becton Dickinson AG), and 0.1% L-cysteine (Sigma-Aldrich chemical)) at 37°C for up to 72 h. *Escherichia coli *strains were grown in Luria-Bertani (LB) broth or on LB agar at 37°C. Antibiotics (ampicillin, 100 μg/mL) were added for selection of *E. coli *carrying recombinant plasmids.

**Table 1:** *Clostridium chauvoei *strains used in this study

Internal reference	Strain	Description and origin
JF1863	ATCC10092	type strain
JF2697	MT	blackleg outbreak, Brazil, 2002
JF3703	595E	acute blackleg, New Zealand, 1959
JF4135	C6 O/D1126/04	acute blackleg, Switzerland, 2004
JF4251	AN2548/02	blackleg outbreak, Sweden, 2002
JF4252	AN1717/06	blackleg outbreak, Sweden, 2006
JF4253	AN2500/07	blackleg outbreak, Sweden, 2007

DNA from *C. chauvoei *strains was extracted by the guanidium thiocyanate method [24]. Ligation, subcloning, plasmid extraction of the DNA fragments and agarose gel electrophoresis (0.7%) were performed as described [25]. Plasmid extraction from recombinant *E. coli *clones was done by alkaline lysis using a Miniprep kit (PeqLab Biotechnologie GmbH, Erlangen, Germany). Sequence analysis was performed using an ampli-*Taq *FS dye terminator kit (Applied Biosystems, Foster City, CA, USA) and the universal primers complementary to the T3 and T7 promoters flanking the multiple cloning site of pBK-CMV, or primers that were derived from obtained DNA sequences.

Comparisons of DNA sequences and their corresponding amino acid (aa) sequences with DDBJ/EMBL/GenBank databases were performed using BLAST analysis [26]. The molecular mass and theoretical isoelectric points (pI) of the sialidase were calculated using ProtParam [27]. Pattern and profile searches were carried out at the online servers Motif Scan [28] and InterProScan [29]. The presence and location of signal peptide cleavage sites in the amino acid sequence were predicted with the program SignalP 3.0 [30]. Prediction of transmembrane helices and topology of the sialidase was carried out with the programs HMMTOP 2.0 [31] and Phobius [32]. The neighbour joining tree of aa sequences from clostridial sialidases was constructed with multiple amino acid sequence alignment by ClustalW2 [33].

### Production of rabbit serum directed against proteins secreted by *C. chauvoei*

A culture (100 mL) of *C. chauvoei *strain JF1863 (Table 1; type strain ATCC10092) grown for 72 h in TGY medium was centrifuged at 9000 × *g *at 4°C for 20 min. Proteins in the supernatant were precipitated overnight at 4°C upon addition of 70% (w/v) ammonium sulphate. Precipitated proteins were sedimented by centrifugation for 10 min at 12000 × *g*. The pellet was suspended in 10 mL of dialysis buffer (0.1 M Na-bicarbonate, pH 8.3, 0.25 M NaCl) and then dialyzed overnight at 4°C with gentle stirring. Aliquots of 500 μL containing 200 μg of dialyzed proteins or 50 μg of purified recombinant protein were mixed with the same quantity of Gerbu adjuvant (Gerbu Biotechnik GmbH, Gaiberg, Germany) for immunizing rabbits three times at intervals of two weeks. Rabbits were bled two weeks after the last immunization and all serum samples were then incubated for 30 min at 56°C to inactivate complement.

### Construction of a genomic library, cloning of the *nanA *gene and DNA sequence analysis

Genomic DNA of *C. chauvoei *strain JF4135 (other name: C6 O/D1126/04; Table 1), a strain that caused acute blackleg in Switzerland in 2004, was partially digested with *Sau*3AI and fragments from 2 to 4 kb were selected to construct a genomic library, using *Bam*HI-digested λ-ZAP-express vector arms, which was packaged with the Gigapack-11 packaging system (Stratagene, La Jolla, CA, USA). The phage library was plated using standard protocols on the *E. coli *strain XL-1 Blue MRF'. Screening of transformed *E. coli *was performed with rabbit serum against secreted proteins of *C. chauvoei*, raised as described above, used at a dilution of 1:100. Positive clones were amplified and finally subjected to in vivo excision using the f1 helper phage in *E. coli *strain XLOLR to obtain phagemid clones in expression vector pBK-CMV. A clone that strongly reacted with the rabbit hyper-immune serum was revealed to contain a 1.7 kb fragment whose sequence showed similarity to the sialidase gene of *Clostridium septicum *[34], as determined by BLAST analysis. The remaining part of the sialidase gene, named *nanA *(neuraminidase A), was detected by screening the same genomic DNA library by DNA:DNA hybridization with a 512 bp digoxigenin-labelled probe amplified from the cloned 1.7 kDa fragment with primers digSiaCC_605F (TTACTAGTGGAAATGGTG) and digSiaCC_1117R (CTTTTGCTGTAGTTTCAC). Digoxigenin labelling of the probe was done according to the manufacturer's instructions (Roche Diagnostics, Rotkreuz, Switzerland). Upon analysis of few clones harbouring partial sequences of *nanA *by primer walking with an ABI Prism model 3100 genetic analyzer (Applied Biosystems), the complete DNA sequence of the cloned gene and the sequences upstream and downstream were determined. Sequence alignment and editing were performed using the software Sequencher 3.0 (GeneCodes, Ann Arbor, MI, USA). The full length *nanA *gene of strain JF3703 (other name: 595E; Table 1), a highly virulent strain isolated in New Zealand in 1959, was determined by PCR amplification and DNA sequence analysis using the same primers as for strain JF4135. The sequence data of the sialidase gene *nanA *have been deposited in the EMBL Nucleotide Sequence Database (European Bioinformatics Institute, Cambridge, UK) under accession numbers FM213081 for strain JF3703 and FM213082 for strain JF4135.

### PCR analysis

The presence of the *nanA *gene of *C. chauvoei *was demonstrated in all seven *C. chauvoei *strains examined (Table 1) by PCR using the primers SiaCC_1371F (ATCAGCAATAGATACATC) and SiaCC_1789R (TGACCTCTTCCTGGTCCTGT). PCR was carried out in 30 μL reaction mixtures containing 1 × reaction buffer B (supplied with FIREPol^® ^DNA polymerase), 2.5 mM MgCl_2_, 0.4 mM of each primer, 1 mM dNTPs, and 2.5 U of FIREPol^® ^polymerase (Solis BioDyne, Tartu, Estonia). Approximately 100 ng of genomic DNA was added as template. Cycling conditions on a 9800 Fast Thermal Cycler (Applied Biosystems) were 3 min denaturation at 94°C, followed by 35 cycles at 94°C for 30 s, 56°C for 30 s and 72°C for 60 s. A final extension step for 7 min at 72°C was included. Amplicons were purified with the High pure PCR product purification kit (Roche Diagnostics).

### Cloning and expression of the CBM40 module of the *C. chauvoei *sialidase and production of antibodies

To generate polyclonal antibodies directed against the CBM40 sialic acid binding domain of NanA, the coding region from nt position 367 to 1389 of the *nanA *gene (corresponding to aa 123 to 463), which also includes the first Arg residue and the RIP (Arg-Ile-Pro) motif of the catalytic site of sialidases, was amplified from *C. chauvoei *strain JF4135 using the primers Sia40_NcoIF (ggtcccatggGTATAAAAGAATACAAAATTTATGCG) and Sia40_HindIIIR (cccaagcttGATGTATCTATTGCTGATGATGCTCC) (nucleotides in lower case are extensions and underlined nucleotides are recognition sites for the restriction enzymes *Nco*I or *Hin*dIII). The resulting PCR product was cloned into the pGEM-T easy vector (Promega Corp., Madison, WI, USA) and transformed into *E. coli *strain DH5α. Recombinant plasmids with the correct sequence were then digested with *Nco*I and *Hin*dIII, and the DNA fragments were inserted into the expression vector pETHIS-1 [35]. The cloned insert was sequenced to ensure correct fusion with the vector's poly-His codons before being transformed into *E. coli *BL21(DE3) cells [36] for expression. Induction and subsequent purification of the recombinant 40 kDa His-tagged protein was performed as described [37]. Purified 40 kDa His-tagged protein was used to immunize rabbits as described above.

### SDS-PAGE and immunoblotting

Pellets and supernatants of *C. chauvoei *strains (Table 1) incubated at 37°C under anaerobic conditions in TGY medium for 72 h were examined by sodium dodecyl sulfate-polyacrylamide gel electrophoresis (SDS-PAGE) and immunoblotting on nitrocellulose membranes (Bio-Rad Laboratories, Hercules, CA, USA). Proteins were separated on 12% acrylamide gels by SDS-PAGE under denaturing or non-denaturing conditions [38]. Non-denaturing gel electrophoresis was performed in the absence of reducing agents. In order to detect the sialidase, the immunoblot membranes were incubated with rabbit polyclonal anti-sialidase 40 kDa peptide serum (diluted 1:1000), followed by incubation with a phosphate- or peroxidase-conjugated goat anti-rabbit IgG heavy and light chains (Kirkegaard & Perry Laboratories, Gaithersburg, MD, USA) diluted 1:2000.

### Expression of functional NanA in *E. coli *DH5α

To express enzymatically active recombinant NanA, we amplified the part of the *nanA *gene from nt position 364 to 2316 (corresponding to aa 122 to 772), encoding CBM40 (sialic acid binding module) and the enzymatically active domain but not CBM32 (Figure 1), from *C. chauvoei *strain JF4135 using the primers SiaCC_BssHIIF (ttggcgcgcAATGATAAAAGAATACAAAATTTATGCG) and SiaCC_HindIIIR (cccaagcttTATAAATTTCCATTTTCTGTTATTAAACC) (nucleotides in lower case are extensions containing recognition sites for the restriction enzymes *Bss*HII and *Hin*dIII, respectively, that are underlined). The resulting PCR product was cloned into the vector T-easy (Promega Corp.) and transformed into *E. coli *strain DH5α. Recombinant plasmids with the correct sequence were then digested with *Bss*HII and *Hin*dIII, and the DNA fragment was ligated into the multiple cloning site of vector pFastBac1 (Invitrogen, Carlsbad, CA, USA) digested with *Bss*HII and *Hin*dIII. Vector pFastBac1 was chosen in order to express active NanA in both prokaryotic and eukaryotic cells, such as insect cells, utilizing the baculovirus expression system. This plasmid harbours the polyhedrin promoter from the baculovirus, which was shown to also have an adequate ability for initiating expression of introduced heterologous genes in *E. coli *[39]. *E. coli *DH5α was transformed with pFastBac1::*nanA *and with empty vector pFastBac1. Sialidase activity of recombinant *E. coli *was measured using the sialidase spot test described below.

### Sialidase activity tests

The fluorogenic substrate 2'-(4-methylumbelliferyl)-α-d-*N*-acetylneuraminic acid (MU-Neu5Ac) (Sigma-Aldrich chemical) was used to assay sialidase activity in a spot test [40] and in a fluorimetric determination in culture fluids [41,42].

For the spot test, a working solution of 15 μM MU-Neu5Ac in 0.17 M sodium acetate buffer (pH 6.5) was prepared. Either colonies of *C. chauvoei*, recombinant *E. coli*, or 10 μL of supernatant from liquid *C. chauvoei *cultures were spotted onto 1-mm Whatman filter paper that had been previously moistened with the MU-Neu5Ac working solution, and then incubated at 37°C for 15 min. The reactions were stopped (0.085 M glycine, 0.2 M sodium carbonate, pH 9.4), the filter papers air-dried and fluorescence observed under a UV light (360 nm excitation wavelength). Observable bright blue fluorescence was recorded as positive. Anti-sialidase 40 kDa peptide serum or pre-immune serum (both diluted 1:10) obtained from the same rabbit were spotted onto the filter paper, at some distance from the bacterial spots but still partially overlying them, to assess the neutralizing effect of the anti-sialidase serum against sialidase activity.

For the fluorimetric determination of culture fluid sialidase activity, strain JF4135 was grown anaerobically for 1 day at 37°C. After cell counting (OD_600 nm_), the culture was centrifuged at 2000 × *g *for 10 min and the supernatant was centrifuged again at 15000 × *g *for 15 min to obtain cell-free supernatant. The reaction was performed in a total volume of 100 μL containing 30 μL of 0.1 M sodium acetate buffer (pH 6.0), 10 μL of a 1 mM MU-Neu5Ac solution in water and 60 μL of sample (each sample consisted of 54 μL cell-free supernatant pre-incubated for 30 min at 37°C with 6 μL of anti-sialidase or pre-immune serum (at several dilutions) prior to fluorimetric quantification of sialidase activity). Samples were incubated at 37°C for 60 min and quantification of sialidase activity was carried out kinetically (at 30 sec intervals) in triplicate with an FLx800 fluorescence microplate reader (BioTek, Luzern, Switzerland). Liberated 4-methylumbelliferone (MU) was quantified at 360/40 nm excitation and 460/40 nm emission in relation to a calibration curve obtained with MU standards (serial 2-fold dilutions ranging from 0 to 100 μM).

## Results

### Characteristics of the *Clostridium chauvoei *sialidase

The full length sialidase gene *nanA *of *C. chauvoei *strain JF4135 consists of 2319 bp. A strong ribosome binding site (AGGTGG) is found eight bases upstream of the putative start codon ATG and is preceded by a potential clostridial promoter sequence including a TATA box (TATAAAAT) and a -35 element (TTTACA) (Figure 1). The NanA protein derived from the nucleotide sequence consists of 772 aa, with a predicted molecular mass of 84.0 kDa and a pI of 4.89. The aa sequence derived from the DNA sequence reveals a leader sequence peptidase cleavage site between position 26 and 27 (Ile-Tyr-Ala↓Asp-Ile), which is characteristic for secreted proteins of Gram-positive bacteria. The putative mature sialidase consists of 746 aa with a calculated molecular mass of 81.1 kDa. At its N-terminal end, aa residues 42 to 186 (numbering always includes the aa residues of the precursor) form the CBM32 module (Figure 1). In the central portion of NanA, aa 190 to 370 form the CBM40 module. The enzymatically active site ranging from aa 384 to 770 contains the usual catalytic residues, common to all sialidases: Tyr-728 (catalytic nucleophile), Arg-400, Arg-613 and Arg-693 (forming the arginine triad, whereas Arg-400 is contained in the RIP motif), Trp-577 (that interacts with the glycerol side chain of sialic acid) and Asp-582 (that forms H-bonds with the four OH-groups of sialic acid). Moreover, the four "Asp-boxes" are found at aa positions 437-444, 570-577, 634-641 and 707-714 (Figure 1).

**Figure 2:**
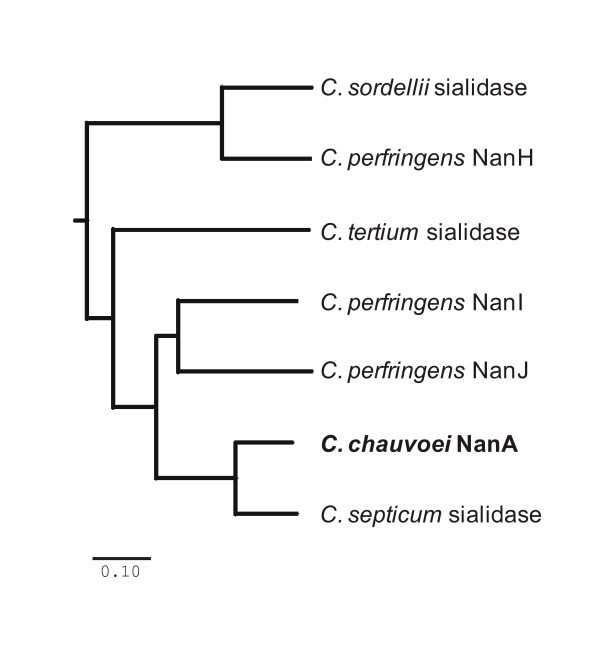
**Neighbour joining tree of clostridial sialidases**. The tree was constructed by ClustalW2 with gap open penalty of 10 and gap extension penalty of 0.05. The bar (0.10) represents substitutions per site. The following sialidase amino acid sequences were aligned: *C. chauvoei *(strain JF4135; this study), *C. tertium *(GenBank: CAA69951), *C. sordellii *(GenBank: AAB38298), *C. septicum *(GenBank: CAA44916), *C. perfringens *NanH (GenBank: P10481), *C. perfringens *NanI (GenBank: NP_561641) and *C. perfringens *NanJ (GenBank: NP_561469).

The aa sequence of NanA from strain JF3703 differs from that of JF4135 at 18 aa loci that do not affect catalytic domains or "Asp-boxes". Amino acid similarities to NanA were found with several clostridial sialidases (Figure 2). Most closely related is the sialidase from *C. septicum *with an aa homology of 79% and 82% identical nucleotides at the gene level.

### Sialidase activity and presence of *nanA *in *C. chauvoei *strains

**Figure 3:**
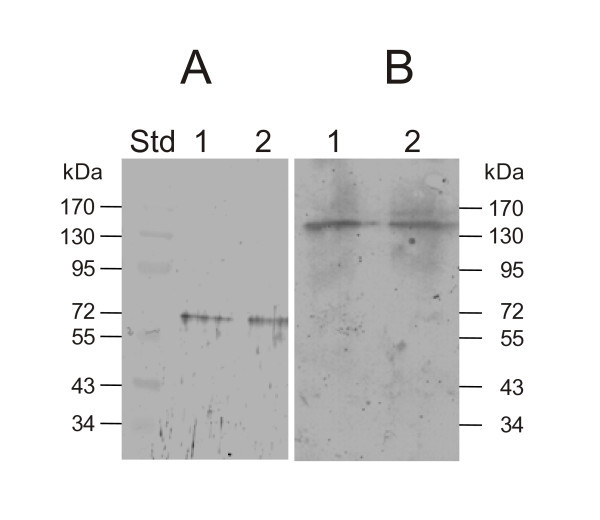
**Dimer form of NanA**. Western blot with anti-sialidase 40 kDa peptide serum under denaturing conditions (panel A) showing the 72 kDa monomer, or under native conditions (panel B) showing the 150 kDa dimer. Lane 1, *C. chauvoei *strain JF3703; lane 2, *C. chauvoei *strain JF4135.

All seven *C. chauvoei *strains used (Table 1) contained the *nanA *gene as revealed by PCR. Sequencing of the amplicons revealed minor nucleotide changes that do not affect however the size of expressed proteins. Monospecific polyclonal antibodies directed against the recombinant 40 kDa peptide representing the CBM40 domain of NanA (Figure 1) reacted on standard immunoblots containing supernatants of *C. chauvoei *cultures of all strains with a 150 kDa and to a lesser extent with a 72 kDa band (Figure 3 shows the results for strains JF3703 and JF4135). The latter band was somewhat smaller than the predicted molecular mass of 81 kDa for mature NanA calculated from the DNA sequence of the *nanA *gene. When non-denaturing gels were used for the immunoblots, only the 150 kDa band was detected (Figure 3), indicating that the *C. chauvoei *sialidase is a 150 kDa dimer of the 72 kDa NanA polypeptide.

**Figure 4:**
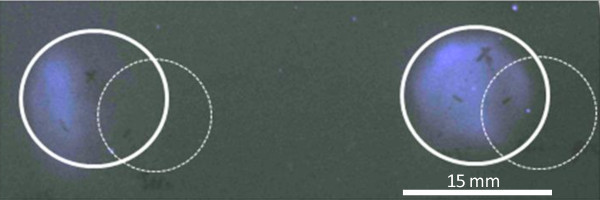
**Sialidase neutralization spot test**. The solid rings show the diffusion zone of the culture supernatant of *C. chauvoei *strain JF4135. The dotted rings show the diffusion zone of: left, anti-sialidase 40 kDa peptide serum; right, pre-immune serum from the same rabbit.

All strains were positive in the sialidase spot test, showing the same spot diameter. Sialidase activity was observed directly with bacterial colonies spotted onto the filter, as well as with supernatant of *C. chauvoei *cultures grown in TGY medium (Figure 4), showing that sialidase was secreted. Moreover, when part of the *nanA *gene encoding the lectin-like sialic acid binding module CBM40 and the enzymatically active domain, but not CBM32, was cloned into plasmid pFastBac1 and transformed into *E. coli *strain DH5α, sialidase activity was found in culture supernatant of the recombinant *E. coli *strain harbouring the plasmid pFastbac1::*nanA *but not in the *E. coli *control strain harbouring the empty vector pFastBac1 only (not shown). Sialidase activity secreted by all *C. chauvoei *strains used in this study and by recombinant *E. coli *was neutralized by monospecific polyclonal antiserum raised in rabbit against the recombinant 40 kDa peptide (Figure 4 shows the results for strain JF4135), showing the functional impact of the CBM40 module covered by this peptide.

**Figure 5:**
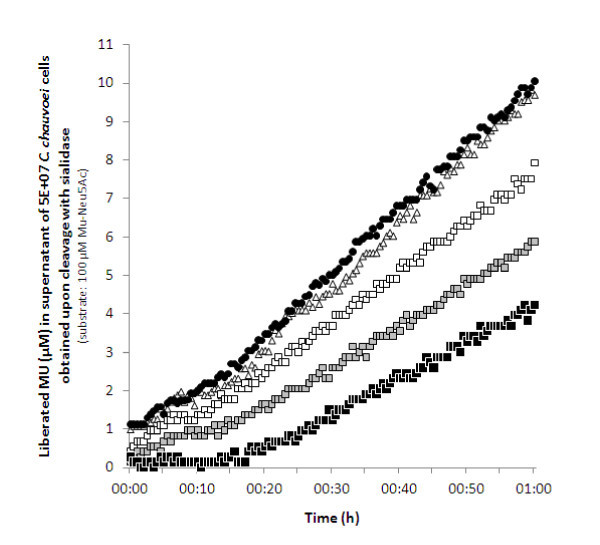
**Fluorimetric determination of culture fluid sialidase activity**. Cell-free supernatant of *C. chauvoei *strain JF4135 was pre-incubated with different dilutions of anti-sialidase 40 kDa peptide serum or pre-immune serum from the same rabbit. Liberated 4-methylumbelliferone (MU) obtained upon cleavage of substrate MU-Neu5Ac with sialidase in culture fluids of 5 × 10^7 ^*C. chauvoei *cells was recorded at 30 sec intervals. Black circles, no pre-incubation with antibodies; white triangles, pre-incubation with pre-immune serum (diluted 1:10); squares, pre-incubation with anti-sialidase serum (white, diluted 1:1000; gray, diluted 1:100; black, diluted 1:10). Plots are the mean of three kinetic measurements of sialidase activity.

Similarly, the anti-sialidase 40 kDa peptide serum had an inhibitory effect on the sialidase activity in culture supernatants of *C. chauvoei *as determined by a kinetic fluorimetric assay (Figure 5 shows the results for strain JF4135). The assay monitored the cleavage of the glycosidic linkage of 100 μM of substrate MU-Neu5Ac upon incubation at 37°C for 60 min with *C. chauvoei *culture fluids. While the culture fluid of 5 × 10^7 ^cells was able to release approximately 10 μM MU from the substrate, anti-sialidase serum could inhibit this activity by 60% when diluted 1:10 (Figure 5). Anti-sialidase serum diluted 1:100 and 1:1000 inhibited the activity by 40% and 20%, respectively. As expected, pre-immune serum from the same rabbit, used as a control at different dilutions, did not affect the *C. chauvoei *culture fluid sialidase activity.

## Discussion

Due to the rapid progression and severity of blackleg, it is appropriate to assume that potent toxins are responsible for the rapid spread of *C. chauvoei *and for the severe lesions that are produced by the pathogen in the target tissues. It has previously been hypothesized that sialidase may have an important role in blackleg by aiding the spread of the bacterium through the tissue [5]. Furthermore, sialidases from *S. pneumoniae*, *P. aeruginosa *and *C. canimorsus *have been demonstrated to be crucial for infection and colonization of the host [16-19]. From these data, it can be hypothesized that the sialidase of *C. chauvoei *might have an important role in the early stage of blackleg infection by degrading tight junctions upon cleavage of sialic acid from mucins and glycoproteins in the infected tissue. Cleavage of sialic acid results in decreased rigidity of the cell surface, thereby facilitating cell motility and thus rendering the target site vulnerable to massive attack by the pathogen that rapidly spreads in the infected tissue. Then, the production of toxins such as hyaluronidases (gamma-toxins), deoxyribonucleases (beta-toxins) and oxygen-labile hemolysins by these bacteria may give rise to necrosis of the infected muscles and alteration of vascular endothelium, both known as characteristic signs of blackleg [5].

Sialidase activity of *C. chauvoei *was reported previously [21] but the corresponding enzyme was characterized only biochemically and not genetically [22,23]. Our genetic and biochemical data reveal a sialidase, named NanA, in *C. chauvoei *that is a 150 kDa homodimer composed of two identical polypeptides of an apparent molecular mass of 72 kDa. Differences between the predicted size calculated from the nucleotide sequence of *nanA *(81 kDa) and the apparent size observed by SDS-PAGE (72 kDa) have previously been reported for several bacterial sialidases, such as *S. pneumoniae *[43,44], *Clostridium tertium *[45] and *C. septicum *[46]. Native sialidase enzymes from bacteria are described as multimers, mostly dimers, of polypeptides having molecular masses ranging between 50 and 80 kDa [45], which agrees with our data for the sialidase of *C. chauvoei*. Although the 300 kDa sialidase described by Heuermann et al. [22] might represent a multimer of the 72 kDa NanA protein, we did not detect such a protein on our immunoblots. The sialidase NanA of *C. chauvoei *is most closely related to the sialidase of *C. septicum *and to NanJ and NanI of *C. perfringens *(Figure 2), the structures of which have been studied in detail [8,10]. In this respect, it is interesting to note that *C. chauvoei *and *C. septicum *are phylogenetically very closely related [47] and at one time were classified phenotypically as the same species [48].

The *C. chauvoei *sialidase NanA displays the same motifs as the *C. perfringens *sialidase NanJ [10]. NanA has a CBM32, or F5/8 type C domain, located at position 42 to 186, which is absent in *C. perfringens *NanI [8,10]. This domain does not seem to be essential for enzyme activity as demonstrated by the sialidase activity of the recombinant NanA without CBM32. This is in agreement with recent data that classify the CBM32 module as a special domain that is involved in binding complex sialic acid substrates containing galactose residues [9,11]. The lectin-like sialic acid binding module CBM40 and the enzymatic active domains show strong similarities to NanI and NanJ of *C. perfringens*. The latter domain contains all the characteristic residues necessary for sialidase activity, as seen in all prokaryotic and eukaryotic sialidases [11], including the catalytic arginine triad with the RIP motif on the first of the three Arg residues, and the characteristic "Asp-boxes". Antibodies directed against the recombinant 40 kDa peptide that includes the entire CBM40 module and part of the enzymatically active domain of NanA, in particular the RIP motif (Figure 1), neutralized the secreted sialidase activity of *C. chauvoei *strains (Figures 4 and 5), confirming their importance in sialidase activity. Furthermore, this result suggests that NanA is the sole or predominant secreted sialidase of *C. chauvoei*. If other sialidases antigenically similar to NanA were present in *C. chauvoei*, supplementary protein bands would have been detected on immunoblots using the same anti-serum against the 40 kDa peptide, but no such additional sialidases were observed (Figure 3).

Since prevention of contact of ruminants with the endospores of *C. chauvoei *that are present in the contaminated soil is virtually impossible, vaccination is the only preventive measure to effectively control blackleg. Current vaccination strategy against this disease relies on the use of a suspension for injection containing formaldehyde-inactivated cultures of virulent strains of *C. chauvoei *and culture supernatant, generally presented in polyvalent formulations. These vaccines are efficient but the particular antigens necessary to induce protective immunity against blackleg are unknown, thus requiring expensive work-intensive animal models for potency testing of vaccine batches. Moreover, molecular quality control of current vaccines, design of novel vaccines and diagnostic tests are mainly hampered by the lack of basic knowledge on the molecular mechanisms of pathogenicity of *C. chauvoei*. Studies on the role of sialidases of *Streptococcus pneumoniae *in otitis media in children and of *Propionibacterium acnes *in acne vulgaris have shown that sialidases of these species induce antibodies that inhibit propagation of bacteria in the host tissues [17,49,50]. When challenged, chinchillas vaccinated with recombinant sialidase from *S. pneumoniae*, expressed in *E. coli*, showed significantly reduced colonization and middle ear invasion compared with non-vaccinated individuals. Taken together, these findings in animal studies and the data of the present work, whereby antibodies raised against the sialidase 40 kDa peptide of *C. chauvoei *effectively neutralized the activity of the infectious agent, indicate that sialidase of *C. chauvoei *may be a good vaccine candidate to protect ruminants against the pathogen and to prevent the occurrence of blackleg. Furthermore, we expect that a combination of this antigen with one or more polypeptides derived from the toxins produced by *C. chauvoei *may have a potentiated protective effect against blackleg.

## Competing interests

The authors declare that they have no competing interests.

## Authors' contributions

EMV and AJ made equivalent contributions to the study. EMV carried out the fluorimetric determination of culture fluid sialidase activity, devised the cloning strategies and participated in the sequence analysis. He drafted the manuscript. AJ constructed and screened the genomic library. He carried out the molecular biological studies and was responsible for expressing the recombinant sialidases as well as testing the resulting polypeptides in immunoblotting and sialidase activity spot tests. YS participated in sequencing and in the immunoassays. KR participated in the design of the study. JF conceptualized the study and supervised all facets of the research. All authors read and approved the final manuscript.
